# Remote OSCE Experience: What First Year Pharmacy Students Liked, Learned, and Suggested for Future Implementations

**DOI:** 10.3390/pharmacy9010062

**Published:** 2021-03-18

**Authors:** Amanda Savage, Lana M. Minshew, Heidi N. Anksorus, Jacqueline E. McLaughlin

**Affiliations:** 1 UNC Eshelman School of Pharmacy, University of North Carolina at Chapel Hill, Chapel Hill, NC 27599-7355, USA; minshew@live.unc.edu (L.M.M.); hanksoru@email.unc.edu (H.N.A.); jacqui_mclaughlin@unc.edu (J.E.M.); 2 Center for Innovative Pharmacy Education and Research, UNC Eshelman School of Pharmacy, University of North Carolina, Chapel Hill, NC 27599-7355, USA

**Keywords:** OSCE, telehealth, remote, student perceptions

## Abstract

During the wake of the COVID-19 pandemic, many schools quickly transitioned their teaching and assessment strategies to online formats. In Spring 2020, a 3-station remote Objective Structured Clinical Examination (OSCE) was implemented for first-year pharmacy students. The day following the remote OSCEs students answered three open-text prompts about the remote OSCE experience: (1) “I liked...”, (2) “I learned…”, and (3) “I suggest…”. Responses were open-coded and frequency counts were calculated to determine the most prevalent codes. Concept maps were created to visualize and explore connections between the codes. Out of 157 students, 156 students completed the reflection assignment, a 99.36% response rate. The three major themes in the Liked data were: Logistics (*n* = 65, 41.7%), Differences In-person Versus Remote (*n* = 59, 37.8%), and Skill Development (*n* = 43, 27.6%). The three major themes in the Learned data were: Technology (*n* = 66, 42.3%), Communication (*n* = 58, 37.2%), and Skill Development (*n* = 56, 35.9%). The three major themes in the Suggest data were: Logistics (*n* = 89, 57.1%), Technology (*n* = 31, 19.9%), and Continuation of Remote OSCE (*n* = 31, 19.9%). Overall, the remote OSCE experience was well-received, and students described it as applicable to their future pharmacy practice. Future work should explore the design, implementation, and outcomes of remote OSCEs.

## 1. Introduction

The Objective Structured Clinical Examination (OSCE) is widely used in schools of pharmacy across the United States and internationally [1]. Harden defined the OSCE as an “approach to the assessment of clinical competence in which the components of the competence are assessed in a planned or structured way with attention being paid to the objectivity of the examination” [2]. OSCEs are a valued form of performance-based assessment in health sciences education because the focus is on evaluating both clinical skills and knowledge and typically involves students rotating through a series of stations where one or two aspects of competence are assessed at each station [2]. 

In the spring of 2020, during the wake of the COVID-19 pandemic, many schools and universities quickly transitioned their teaching strategies to remote online learning environments. This transition necessitated assessments such as OSCEs to also transition to remote online formats. Several scholarly blog posts described the operational processes and challenges encountered by pharmacy educators administering remote OSCEs by utilizing video-teleconferencing (VTC) programs. Lucas and Forrest, for example, highlighted the challenge of assessing non-verbal communication due to the limitations of VTC platforms [3]. Technology challenges such as connectivity issues can further complicate remote OSCE implementation [4]. However, none explored or described student perceptions of remote OSCE experiences. Student perceptions are influenced by many factors and have diverse effects in educational settings [5]. Thus, gaining insight into student perceptions provides valuable information regarding the design quality of the curricular innovation. 

Since OSCEs target the assessment of clinical skills in trainees, the design of remote OSCEs mirror telehealth, defined by the US Department of Health and Human Services as “the use of electronic information and telecommunications technologies to support and promote long-distance clinical health care, patient and professional health-related education, and public health and health administration. Technologies include videoconferencing, the internet, store and-forward imaging, streaming media, and landline and wireless communications” [6]. A brief literature search using the key words “telehealth” and “OSCE” and/or “student perceptions of telehealth OSCE” revealed a sparsity of information related to telehealth simulations or OSCEs for pharmacy learners. Skoy and colleagues revealed no difference between students’ abilities to successfully provide medication consultation to patients between face-to-face and telehealth encounters [7]. However, students in this study agreed that there were differences in “difficulty, stress, interpreting patient cues, and in maintaining eye contact and tone of voice between the two means of consultation” [7]. Recently, VanLangen and colleagues found virtual skills-based assessments to be an appropriate mechanism to assess student communication skills; however, they did not observe a strong preference for virtual skills-based assessments in the future [8]. Similar research from nursing and medicine describes telehealth OSCE simulation yet fails to explore student perspectives [9,10]. 

Considering the significant need for telehealth during the COVID-19 pandemic, determining strategies to teach and assess students’ ability to provide patient care utilizing remote OSCEs is timely. The Center for the Advancement of Pharmaceutical Education (CAPE) 2013 Educational Outcomes outline that students should be effective communicators and utilize available technology and other media to provide care to patients [11]. Additionally, the American Society of Health-System Pharmacists’ position on telepharmacy states, “appropriately trained and equipped pharmacists” should apply telepharmacy to “suitable functions of pharmacy operations and patient care to improve patient outcomes, expand access to healthcare, and enhance patient safety” [12]. The purpose of this study was to explore student perceptions following implementation of a 3-station remote OSCE administered during spring of 2020 and utilize the feedback to develop strategies for future remote OSCE implementations.

## 2. Materials and Methods

### 2.1. OSCE Design

Three remote OSCE stations administered in the spring of 2020 at the Eshelman School of Pharmacy at the University of North Carolina at Chapel Hill were designed to simulate a telehealth encounter with a patient or provider. The OSCE stations were administered over two days, one week apart, and included conducting a medication history interview on Day 1 and presenting a patient case to a pharmacist preceptor and providing medication education to a patient on Day 2. Students were scheduled in intervals to provide sufficient time to conduct the encounter, plus a buffer was built into each interval to allow for troubleshooting technical difficulties. 

Students were required to create a Zoom meeting link for their individually scheduled OSCE time and post their meeting link and a contact phone number in an assignment via the school’s learning management system (LMS). Students were instructed to “arrive” on Zoom 5 min prior to their scheduled OSCE start time. Detailed student instructions were provided in written format, which included a directive to record the encounter. Postgraduate Year-1 (PGY1) teaching assistants served as the standardized patient/provider as well as the evaluator for each encounter. After the encounter, students posted their recorded session in the LMS and teaching assistants had access to the recordings to resolve any grading discrepancies. 

### 2.2. Data Collection

The day after the remote OSCEs, students were asked to complete a post-OSCE assignment consisting of self-evaluation and reflection questions related to each of the three OSCE stations. In addition to these questions specific to the OSCE stations, three open-text prompts capturing student perceptions about the remote OSCE experience were included: (1) “I liked....” (Liked data), (2) “I learned…” (Learned data), and (3) “I suggest…” (Suggest data). Open-text responses were chosen over Likert-style survey questions to capture students’ unique ideas and perceptions rather than expressing agreement/disagreement for a given statement. The post-OSCE assignment was administered via Qualtrics survey to the students in the LMS. Students were given six days to complete the post-OSCE assignment, and students were awarded full points (30 points/400 points) for completion, which accounted for approximately 7.5% of the final exam grade. This study focuses on the three open-text prompts to assess student perception of the remote OSCE experience. Data were deidentified prior to data analysis. The research was determined to be exempt by the University’s Institutional Review Board. 

### 2.3. Data Analysis

The analysis of the qualitative data was iterative and consisted of creating inductive codes, using frequency counts to identify the prevalence of student perceptions, and the creation of concept maps to identify connections and condense codes into broader themes [13,14]. Using Excel™, two researchers (AS and LM) met to open code and discuss approximately 32% of the student responses together. Confidence in the consistent application of the codes was achieved through continuous discussion during the open-coding sessions [13,14]. Moving forward, a single researcher (AS) open-coded the remaining Learned data. If a new code emerged during individual coding, it was documented, defined, and then discussed with the other researcher. The second researcher (LM) reviewed the coded responses independently noting additions and deletions of applied codes. Inter-coder agreement for the Learned data (94%) was calculated by following Miles and Huberman’s formula [15]. The researchers traded roles and completed the same process for the Liked data resulting in inter-coder agreement (89%). During the coding of the Suggest data, new codes were continuously added to the codebook, and therefore both researchers coded all student responses together. Throughout the coding process, as new codes emerged, all previously coded data was reviewed for application of new codes [14]. All coding discrepancies were discussed and resolved.

**Figure 1:**
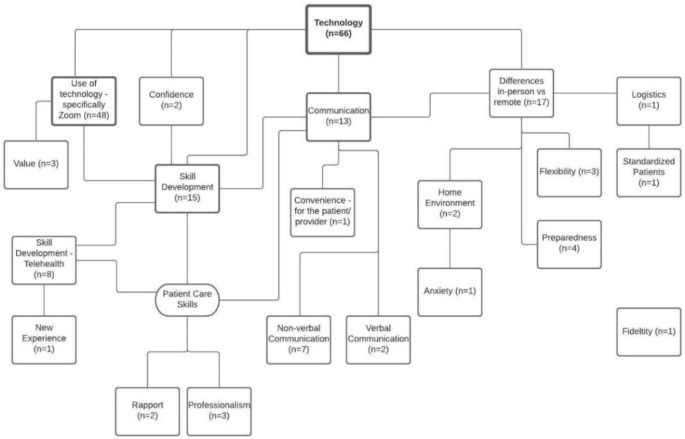
This figure is an example concept map for the Technology theme within the Learned data. Concept maps were created by the researchers to show the connectivity of the various subthemes.

Frequency counts were calculated to determine the most prevalent codes within the data. A single student response could contain multiple different codes; however, a single code was only applied once per student response. Therefore, the frequency count of each code represents one student. For example, “Technology” was coded 66 times in the Learned data, indicating 66 students spoke about Technology in their response. The most frequent codes in each data set were identified, and LucidChart™ (LucidSoftware Inc., South Jordan, UT) was used to create concept maps representing connections between the codes. This allowed the researchers to visualize and explore the connections between the codes and identify broad themes and associated subthemes. An example concept map is shown in Figure 1. 

## 3. Results

Out of 157 students, 156 students completed the post-OSCE assignment, a 99.36% response rate. Results are presented within the three remote OSCE experience prompts Liked, Learned, and Suggest. For each prompt, three major themes and corresponding subthemes are presented. Student quotes are included to illustrate these major themes and connections with subthemes. Table 1 contains rank order of codes and their corresponding frequency counts. 

### 3.1. Liked

The three major themes were: Logistics (*n* = 65, 41.7%), Differences In-person Versus Remote (*n* = 59, 37.8%), and Skill Development (*n* = 43, 27.6%). Student comments about logistics focused on what they liked about the process: receiving materials ahead of time and clarity of instructions provided by course faculty. One student shared, “I liked that we got the patient information ahead of time…being able to prepare ahead of time helped me feel more confident and less stressed about the encounters. Additionally, I was a bit intimidated by setting up a Zoom meeting and recording it, but I thought the instructions we were provided were really helpful and easy to follow.”

Other subthemes in logistics were connected to the efficiency of the process, staying on schedule, and the convenience of conducting the OSCE encounter in students’ home environments. These subthemes were also present within the theme of differences between in-person and remote. One student stated, “I liked how I could be at home and be able to do the OSCE comfortably. The administration of the Zoom OSCEs was very well thought out and carried out. The [teaching assistants] TAs were always on time and provided great instruction during each interaction.” In addition to comments about the home environment, students discussed the differences in the technology platforms used for the remote OSCE versus an in-person encounter. Another student said, “I liked the fact that we got an opportunity to practice Telehealth! With Telehealth becoming more and more prevalent in healthcare, I think it’s important to get used to advancing technology.”

While students acknowledged the differences between an in-person and remote encounter, they also discussed the applicability of this experience in developing skills for their future practice as pharmacists. One student shared, “While there is some benefit to face-to-face interactions, virtual medicine and virtual appointments may be pertinent for our future careers. It was good to practice a different setting and become familiar with online medication counseling/presenting.” Another student responded, “I liked how it gave relevant practice, as we may utilize Zoom or similar platforms in telepharmacy experience in the future.”

### 3.2. Learned

The three major themes in the Learned data were: Technology (*n* = 66, 42.3%), Communication (*n* = 58, 37.2%), and Skill Development (*n* = 56, 35.9%). Within the technology theme, students primarily discussed learning about the functionality of Zoom and how to troubleshoot any technical difficulties. One student shared, “I definitely learned how to use Zoom much more effectively. I had very little experience with it, so this was definitely also good in helping us to navigate the software. I also learned how to adjust on the fly and help trouble shoot problems quickly and effectively.” Another student said, “I learned how to set up a Zoom meeting and how to use Zoom overall. This will be a very valuable skill as many meetings are being set up through Zoom.” Subthemes within technology were closely interconnected with communication and skill development. Another student stated they learned, “how to appropriately handle different types of patient interactions via technology.”

Student responses about skill development can be categorized by patient care skills and non-patient care skills. Non-patient care skills learned were primarily related to technology as discussed above. Patient care skills learned included descriptions of applying the remote OSCE experience to providing care to real patients as a practicing pharmacist. One student stated, “It sometimes is still important to be able to see your patients/see a healthcare provider in person because you do feel a disconnect when doing these activities virtually. I also learned that technology has become so important in our lives and that it’s great that we have technology to assist us in times like these when the alternative may be an inability to see a healthcare provider at all.” Students also described non-verbal and verbal communication skills needed in a remote environment. One student shared, “I learned how giving interviews/educational meetings over Zoom conference has a very different feel to it than doing it in real life. I was surprised how much I rely on patient body language to tell me how well I am communicating with the other person, however, over Zoom it is hard to interpret body language.”

The major themes of Technology, Communication, and Skill Development were very interconnected within student responses. For instance, one student said, “I learned how to effectively communicate with a patient over Zoom and demonstrate active listening during a virtual encounter. I also learned how to troubleshoot any issues with Zoom and improved my problem-solving skills.” 

### 3.3. Suggest

The three major themes were: Logistics (*n* = 89, 57.1%), Technology (*n* = 31, 19.9%), and Continuation of Remote OSCE (*n* = 31, 19.9%).

In the Suggest data, students focused on the logistics of the remote OSCE experience. Logistics was defined as any reference to instructions, process, scheduling, operations, or implementation. Comments on the process and implementation of the remote OSCE were the most common suggestions made regarding logistics. Responses related to the process included the post-OSCE assignment and practice sessions in a remote environment. One student shared, “I would have preferred to have two separate reflections (one for each week) and had the opportunity to reflect on my med hx [medication history] OSCE right after it occurred.” Student suggestions related to implementation included: adding screen sharing of a timer for the encounter and participating in multiple stations sequentially with the same evaluator. Students also discussed the use of standardized patients during the remote OSCE encounter, specifically focusing on the fidelity of the experience. One student shared, “I suggest using real actors in the future. The TAs were good, but it was almost weird interacting with familiar faces. I like working with the actors because they make the entire encounter feel more realistic…”. Finally, some students did not have any suggestions for how to improve the logistics of the remote OSCE and indicated they were pleased with how the experience was conducted. One participant shared, “I suggest nothing. I think the Zoom OSCE was implemented in a way to prepare for most errors that could occur.” 

Many student responses related to logistics were interconnected with technology. Students discussed technical challenges with Zoom, including creating the meeting link, initiating, and uploading their recorded encounter following the remote OSCE. For example, one student said, “I suggest that future students could have additional instructions regarding the ‘Recording Zoom’ feature as well as ‘saving’ the Zoom File.” Several students also commented about the challenges of conducting the encounter when their evaluator did not have their webcam turned on. One student suggested, “asking the graders to turn on their cameras because it felt kind of weird talking to a blank screen in my own room.” Other students mentioned experiencing technical challenges such as internet connectivity issues.

Further, students suggested the continuation of a remote OSCE within future courses. Most students connected continuing the remote OSCE to their skill development, with many students recognizing the value in conducting a remote OSCE. One student suggested “to do a virtual OSCE every year, even outside of a pandemic setting as I have learned that it is important that student pharmacists are able to engage with patients virtually and in person”. However, a few students suggested we not consider conducting remote OSCEs in future courses because they did not see the utility of this encounter in future practice experiences. One student responded, [I suggest] “to make every effort to do OSCEs in person as it is a more valuable experience and prepares us for interactions that we will have in practice better than a virtual OSCE.”

### 3.4. Thematic Connections

There were several recurring themes across the three OSCE experience prompts; however, students described these themes differently based on the lens through which they were viewed (i.e., the prompts). 

For example, the technology code was defined as any general reference to technology: computer, Wi-Fi, software, or reference to technical difficulties. In the Learned data, students described technology in terms of skills needed for patient care, whereas in the Suggest data, students identified their problems with technology during the patient/practitioner interaction. For the learned prompt, one student said, “Through this experience I learned that you can definitely still have meaningful contact and encounters with a patient or other health care professional via video call, and all of the same things still apply, like eye contact, confidence, demeanor, and professionalism.” The same student had the following comment for the suggest prompt: “I think it is important to work out the kinks in a situation like this of course, especially around recording the encounter and uploading to Sakai.” Thus, even though the technology code was applied in both data sets, the resulting student comments expressed different ideas.

Differences in person versus remote (DIPR), defined as the participant acknowledging or comparing differences between completing the encounter in-person vs completing the encounter remotely, was another frequent theme across all three prompts where discussion varied. In the learned data, DIPR comments were connected to technology and skill development. A student shared, “It sometimes is still important to be able to see your patients/see a healthcare provider in person because you do feel a disconnect when doing these activities virtually. I also learned that technology has become so important in our lives and that it’s great that we have technology to assist us in times like these when the alternative may be an inability to see a healthcare provider at all.” The DIPR comments in the Liked data were connected to convenience for the student, convenience for the patient/provider, home environment, and anxiety. For instance, the same student said, “Being able to wear comfortable clothing from the waist down. Also not having to leave my house was a nice thing, which I’m sure in the real world is a sentiment some patients would share. Doing the OSCE virtually also did help to calm my nerves a little since it didn’t feel as high pressure as the setting in [school].” Finally, in the suggest data, DIPR was connected to logistics: “I cannot think of any suggestions at the moment. The OSCEs went pretty smoothly for me. Maybe if there was a way to set up a timer that I am aware of as well so that there is something like the 2-min warning we got for our physical OSCEs.” 

Comments within the Learned data set regarding skill development fell into two categories: non-patient care skills and patient care skills. For non-patient care skills, students frequently noted they learned skills related to technology. However, within the theme of skill development in the Liked data set, students focused on patient care skills and noted they liked the idea of applying the learned skills to future practice. For example, one student commented for the learned prompt that “I learned how to set up a Zoom meeting and how to use Zoom overall. This will be a very valuable skill as many meetings are being set up through Zoom.” Within the liked prompt, the same student said: “I liked the flexibility of the Zoom OSCE. It gave me insight on how tele-health will be like when communicating with rural populations.”

## 4. Discussion

The three prompts of Like, Learned, and Suggest elicited a variety of student responses regarding their remote OSCE experience. Students primarily shared their thoughts about the use of technology, their skill development, the logistics of the remote encounter, and the differences between a remote and in-person OSCE experience with several students describing the remote OSCE experience as applicable to their future pharmacy practice. Students did not receive formal instruction on how to conduct themselves during a telehealth patient encounter; however, students recognized the differences between in-person and remote encounters. The remote OSCE experience appeared to enable students to learn patient care and non-patient care skills. Students identified communication with patients in a teleconference setting and general technology skills, such as how to navigate Zoom, as key things they learned from the experience. Finally, students suggested improvements to the logistics and implementation for future remote OSCEs, such as using standardized patients. Many students believed the initial remote OSCE was an excellent learning experience and recommended that it be continued in the future.

While most students recognized the applicability of VTC to practice, some students failed to associate telehealth with other forms of telecommunication (e.g., phone, email). Students must recognize telehealth as a needed skill in practice and not as a temporary or novel form of practice associated with the COVID-19 pandemic. In 2016, nearly 61% of health care institutions and approximately 50% of hospitals in the United States utilized some form of telehealth [16]. Additionally, telehealth is an important component of providing care to underserved rural and urban populations [17]. Further, communication through relevant technology is a part of the CAPE 2013 Educational Outcomes [11]; thus, it is imperative that students recognize that telehealth, in its various forms, will be something they encounter as a health care professional. A recent commentary by Frenzel and Porter described a call to action to purposefully incorporate tele-education into curricula in order to prepare pharmacy graduates to provide patient care using telehealth [18]. 

There are differences in communication between telehealth and face-to-face patient encounters. In particular, students identified non-verbal cues as essential for proper interaction in a patient encounter yet struggled to read these cues in the remote setting. Skoy et al. explored the comparison of virtual and face-to-face patient encounters and found that students identified differences in “interpreting patient cues, and in maintaining eye contact and tone of voice between the two means of consultation” [7]. To continue remote OSCEs in the future, students may require support developing their verbal and non-verbal communication skills to effectively provide patient care. We suggest pharmacy educators provide support by incorporating strategies for and highlighting the nuances of verbal and non-verbal communication for both face-to-face and telehealth patient interactions in teaching and assessment. 

While the remote OSCE experience went smoothly, some opportunities for process improvement were identified. Supplying sufficient time between sessions, as well as having students provide contact phone numbers, proved to be critical to staying on schedule while allowing for troubleshooting of technical difficulties. Due to the timeline required for the implementation of this remote OSCE experience and the additional training involved, we did not utilize hired actors as standardized patients; rather, course teaching assistants served as both the patient/provider and the evaluator Student feedback revealed a preference for standardized patients, which makes the encounter “feel more real”. Thus, we recommend using standardized patients when time and resources allow to increase the fidelity of the remote OSCE encounter. In addition, we recommend providing detailed written and verbal instructions for students and all parties involved in the implementation of the remote OSCE. Students struggled to upload their recorded sessions due to file size and unfamiliarity with the LMS’s upload procedures. Having a formal document that outlines each step to ensure appropriate file size and upload procedures will make this process easier. Hopwood et al. recently listed tips related to designing and implementing a remote OSCE; these tips included adequate planning, training, piloting, and modification of scenarios to simulate a telehealth encounter [19]. Additionally, they suggested getting detailed feedback from key stakeholders involved in the experience to continue to revise and improve the implementation of the remote OSCE [19]. Our study does this by analyzing and incorporating student perceptions in the revision process of the remote OSCE. 

While our study is an initial step in exploring the promise of remote OSCEs and what they mean for the future of assessments in PharmD curricula, there are a few limitations associated with the current study. First, the remote OSCE was modified and implemented in 10 days; therefore, the initial design of the assessment was rushed. Although this is not a direct limitation to the study, with more time, a different assessment design and implementation strategy could have been generated. Second, the study represents a single school and a single cohort of students; therefore, the findings are not generalizable across schools of pharmacy. However, the study does provide insight into student perceptions of a remote OSCE as well as design and implementation suggestions. Finally, there is a slight probability of social desirability bias due to students including responses they thought their course director wanted to hear. However, students completed the three prompts prior to receiving their OSCE grade, and the prompts were part of a post-OSCE assignment in which students were awarded points for completion. Further, the responses were deidentified by the course director prior to data analysis with a qualitative methodologist not associated with the course. 

## 5. Conclusions

There is limited literature about remote OSCEs, and future work needs to explore the design and implementation of remote OSCEs, student learning outcomes, and student perceptions of the experience. The COVID-19 pandemic forced schools of pharmacy to transition to remote learning and required students in a required first-year skills laboratory course to complete their final OSCE remotely. Although forced, the remote OSCE was generally well-received, with students identifying value in the experience while also sharing process improvement opportunities, and many students suggested that remote OSCEs simulating telehealth encounters be included as future assessments. Students recognized the adjustments made to the assessment in the wake of COVID-19 mirrored the shift to telehealth happening concurrently in pharmacy practice. Thus, we recommend that a telehealth OSCE be incorporated into PharmD curricula as it provides students an opportunity to interact with patients in a new medium, one they will experience in practice. 

## Figures and Tables

**Table 1:** Rank order of code frequencies.

Learned	*N* = 156 (100%)	Liked	*N* = 156 (100%)	Suggest	*N* = 156 (100%)
Technology	66 (42%)	Logistics	65 (42%)	Logistics	89 (57%)
Communication	58 (37%)	Differences in-person vs. remote	59 (38%)	Technology	31 (20%)
Skill development	56 (36%)	Skill development	43 (28%)	Continuation of Remote OSCE	31 (20%)
Differences in-person vs. remote	50 (32%)	Convenience—for the student	42 (27%)	Differences in-person vs. remote	19 (12%)
Use of technology—specifically Zoom	48 (31%)	Skill Development— Telehealth	38 (24%)	Skill development	19 (12%)
Skill Development—Telehealth	24 (15%)	Value	26 (17%)	Skill Development—Telehealth	19 (12%)
Non-Verbal Communication	22 (14%)	Home Environment	25 (16%)	Standardized Patients	18 (12%)
Preparedness	16 (10%)	Anxiety	23 (15%)	Fidelity	16 (10%)
Rapport	15 (10%)	Technology	18 (12%)	Use of technology —specifically Zoom	14 (9%)
Confidence	11 (7%)	Preparedness	15 (10%)	Grading	13 (8%)
Anxiety	10 (6%)	Use of technology —specifically Zoom	12 (8%)	Preparedness	12 (8%)
Verbal Communication	9 (6%)	Standardized Patients	9 (6%)	Value	9 (6%)
Flexibility	8 (5%)	Communication	8 (5%)	Anxiety	7 (4%)
Value	7 (4%)	Flexibility	7 (4%)	Resources	5 (3%)
Professionalism	6 (4%)	Resources	7 (4%)	Communication	4 (3%)
Home Environment	5 (3%)	Confidence	5 (3%)	New Experience	4 (3%)
Convenience—for the student	2 (1%)	Fidelity	5 (3%)	Professionalism	2 (1%)
Convenience—patient/provider	2 (1%)	Convenience—patient/provider	4 (3%)	Rapport	2 (1%)
Logistics	2 (1%)	New Experience	4 (3%)	Confidence	1 (1%)
New Experience	2 (1%)	Grading	2 (1%)	Convenience—patient/provider	1 (1%)
Standardized Patients	2 (1%)	Perspective	2 (1%)	Flexibility	1 (1%)
Anonymity of the internet	1 (1%)	Continuation of Remote OSCE	1 (1%)	Home Environment	1 (1%)
Fidelity	1 (1%)	Non-verbal Communication	1 (1%)	Non-verbal Communication	1 (1%)
Continuation of Remote OSCE	0	Professionalism	1 (1%)	Verbal Communication	1 (1%)
Grading	0	Rapport	1 (1%)	Anonymity of the internet	0
Perspective	0	Anonymity of the internet	0	Convenience—for the student	0
Resources	0	Verbal Communication	0	Perspective	0
No response, N/A	0	No response, N/A	0	No response, N/A	16 (10%)
